# Silage produces biofuel for local consumption

**DOI:** 10.1186/1754-6834-4-46

**Published:** 2011-11-01

**Authors:** Hiroko K Kitamoto, Mitsuo Horita, Yimin Cai, Yukiko Shinozaki, Keiji Sakaki

**Affiliations:** 1 National Institute for Agro-Environmental Sciences, 3-1-3 Kannondai, Tsukuba, Ibaraki 305-8604, Japan; 2 National Institute of Livestock and Grassland Science, Nasushiobara, Tochigi 329-2793, Japan; 3 Research Institute for Innovation in Sustainable Chemistry, National Institute of Advanced Industrial Science and Technology, Tsukuba Central 5-2, Higashi 1-1-1, Tsukuba, Ibaraki 305-8565, Japan; 4 Japan International Research Center for Agricultural Sciences, 1-1 Ohwashi, Tsukuba, Ibaraki 305-8686, Japan

## Abstract

**Background:**

In the normal process of bioethanol production, biomass is transported to integrated large factories for degradation to sugar, fermentation, and recovery of ethanol by distillation. Biomass nutrient loss occurs during preservation and degradation. Our aim was to develop a decentralized ethanol production system appropriate for farm or co-operative level production that uses a solid-state fermentation method for producing bio-ethanol from whole crops, provides cattle feed, and produces no wastes. The idea is to incorporate traditional silage methods with simultaneous saccharification and fermentation. Harvested, fresh biomass is ensiled with biomass-degrading enzymes and yeast. Multiple parallel reactions for biomass degradation and ethanol and lactic acid production are induced in solid culture in hermetically sealed containers at a ranch. After fermentation, ethanol is collected on site from the vapor from heated fermented products.

**Results:**

The parallel reactions of simultaneous saccharification and fermentation were induced efficiently in the model fermentation system. In a laboratory-scale feasibility study of the process, 250 g of freshly harvested forage rice with 62% moisture was treated with 0.86 filter paper units/g dry matter (DM) of cellulase and 0.32 U/g DM of glucoamylase. After 20 days of incubation at 28°C, 6.4 wt.% of ethanol in fresh matter (equivalent to 169 g/kg DM) was produced. When the 46 wt.% moisture was gathered as vapor from the fermented product, 74% of the produced ethanol was collected. Organic cellular contents (such as the amylase and pronase degradable fractions) were decreased by 63% and organic cell wall (fiber) content by 7% compared to silage prepared from the same material.

**Conclusions:**

We confirmed that efficient ethanol production is induced in nonsterilized whole rice plants in a laboratory-scale solid-state fermentation system. For practical use of the method, further study is needed to scale-up the fermentation volume, develop an efficient ethanol recovery method, and evaluate the fermentation residue as an actual cattle feed.

## Background

**Figure 1:**
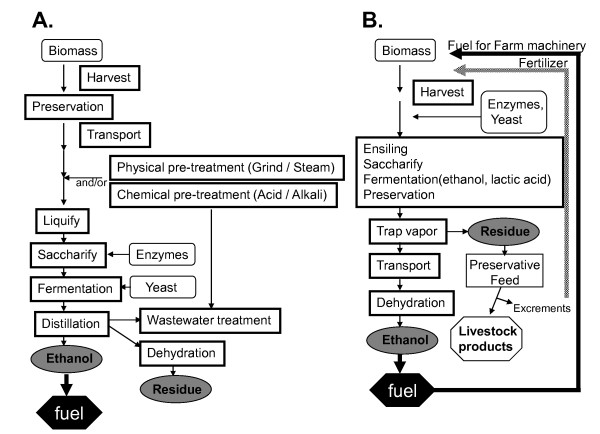
**Processes of ethanol production from cellulosic biomass**. (A) Normal fermentation process; (B) solid-state fermentation system.

Carbohydrate in plants is classified into two main categories, structural and nonstructural compounds. In the normal process of cellulosic bioethanol production, structural carbohydrates are the main carbon source. Tropical grass species with high dry matter (DM) content and lower nonstructural carbohydrate content are good candidate materials for this process [1,2]. Harvested biomass of low bulk density is transported to integrated large factories for pretreatment under acid or alkaline conditions to degrade the structural carbohydrates (Figure 1A). After pretreatment, however, a large amount of enzyme (3.5 to 25 filter paper units (FPU)/g DM of cellulase [3,4]) is still needed for the hydrolysis of cellulose.

Across the world, cattle feed is mainly prepared and preserved as lactic acid fermented crops, so-called silage. Lactic acid kills bacteria that are derived from plant surfaces and the soil and cause the materials to rot. The silage process consistently produces high-quality feed with a minimum of harvesting losses, regardless of weather conditions. This process also should be a suitable method for preserving biomass for fuel production. The content of nonstructural water-soluble carbohydrates (WSC) of crops for silage is dependent on the period of harvest [5]. The main components of WSC in crops for silage are glucose, fructose, and sucrose. Stems also contain easily biodegradable polysaccharides. The WSC contents of cereals used for silage are 80 g/kg DM to 307 g/kg DM in whole corn plant and 46 g/kg DM to 318 g/kg DM in whole barley. During the vegetative stage of growth, soluble sugars are the main nonstructural carbohydrates present in cereals, but these decline after fertilization, and starch content rises during subsequent grain development. The DM content of whole cereal plants used for silage increases with maturity, but the digestibility of whole corn plant remains relatively constant across the plant's developmental stages. Although the nonstructural carbohydrate nutrients of plant materials are much easier to convert to ethanol than cellulose is, nonstructural carbohydrates are solubilized to the wastewater fraction in the normal pretreatment process. If the conversion rate to ethanol is sufficient without pretreatment and the fermented residue is nutritionally sufficient for feed, it may be possible to produce bioethanol without pretreatment, which would lower costs and energy input.

In a previous study, we found that silage made from whole rice plants can be used as material for bioethanol production, and proper selection and combination of commercially available enzymes can induce simultaneous saccharification and fermentation (SSF) without pretreatment [6]. The maximum ethanol yield of 238 ± 29.6 g/kg DM was observed 14 days after treatment of powdered whole rice plants (10% (w/v)) with 8.6 FPU/g of commercially available cellulase from *Acremonium cellulolyticus *(ACS) in 0.1 M sodium acetate buffer (pH 4.5) at 30°C. However, the final ethanol concentration was 2.38 wt.% in the reaction solution, and ethanol recovery requires a lot of energy.

Under anaerobic conditions, yeasts are able to withstand lactic acid better than most other microorganisms using intracellular homeostatic regulation, which involves the formation of ATP with the fermentation of sugar to ethanol [7,8]. Jonsson and Pahlow observed a population of yeasts obtained during ensiling [9]. Under limited fermentative sugar content, the addition of lactic acid bacteria at ensiling brought about competition among the microorganisms for the fermentative sugar in the crop. Lactic acid bacteria consumed fermentative sugar in silage for lactic acid production, and the yeast population was reduced under the acidic condition without fermentative sugar. When preparing silage with the addition of lactic acid bacteria in a crop with sufficient fermentative sugar, however, the population of fermentative yeast is relatively high. A small amount of ethanol is often produced in silage through the fermentation of residual sugar by yeast and/or heterofermentative lactic acid bacteria [5]. When producing silage using crops with low sugar content, or of low digestibility, cellulase may be added at ensiling to release sugar for lactic acid fermentation [10]. Tomoda *et al*. reported the effect of adding ACS to induce lactic acid production during silage fermentation of alfalfa [11]. At ensiling, they added cellulase with 0.089 U/g DM avicelase activity to material that contained 8.81% DM of fermentative sugar with 81% moisture content. After 14 days of fermentation at 26°C, addition of the enzyme induced a lower pH (pH 4.45 versus pH 6.04 in silage without added enzyme) and greater lactic acid production (5.664 wt.% DM versus 0.516 wt.% DM without enzyme).

Based on these traditional, widely used processes, we devised a way to induce ethanol fermentation in silage. In this study, we tested a method of solid-state fermentation of whole crops that controls the spontaneous fermentation of the natural environment to induce the growth of lactic acid bacteria and yeast. The new fermentation techniques could be used for the production of biofuel and forage at the local farm or co-operative level. Our findings indicate the efficiency of fermentation and distillation using the solid-state fermentation system.

## Results and Discussion

### Enzymatic hydrolysis of whole plants in a solid-state fermentation model system

We hypothesized that the inoculation of a greater amount of carbohydrate-hydrolyzing enzymes (such as cellulases, pectinases, and amylases) and ethanol-fermenting yeast at ensiling would induce the degradation of biomass, and the produced sugars would be simultaneously converted to lactic acid and ethanol by lactic acid bacteria and yeast, respectively, during the solid-state fermentation of biomass (Figure 1B).

**Figure 2:**
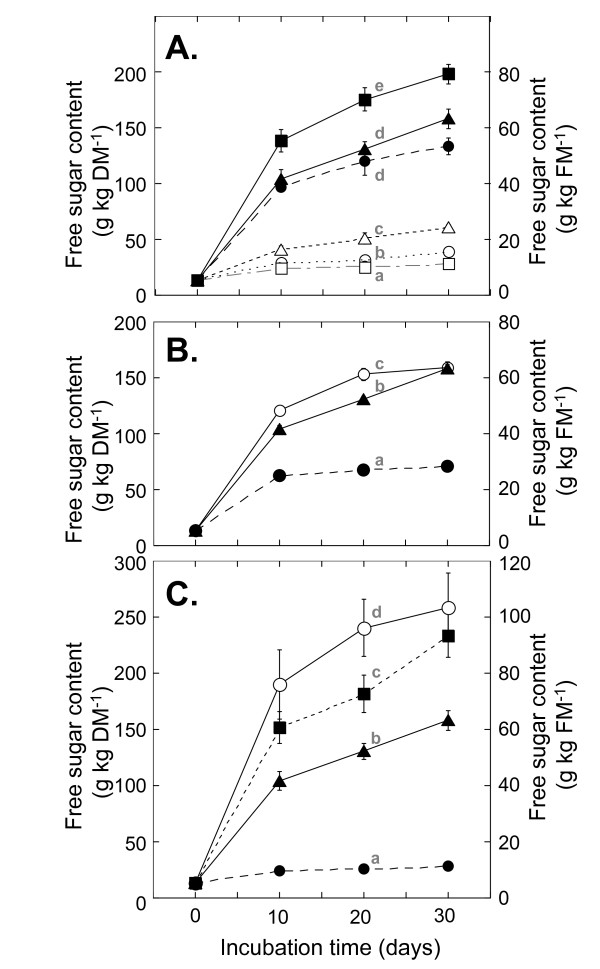
**Enzymatic saccharification of whole rice plants in the solid-state fermentation model system**. **(A) **Free sugar (sum of glucose, fructose, and sucrose) produced from whole rice plants by the addition of various concentrations of ACS cellulase. ACS addition amounts were 1.29 FPU/g DM (black square), 0.86 FPU/g DM (black triangle), 0.43 FPU/g DM (black circle), 0.086 FPU/g DM (open triangle), 0.022 FPU/g DM (open circle), and without enzyme addition (open square). **(B) **Free sugar (sum of glucose, fructose, and sucrose) produced from whole rice plants by the addition of 0.86 FPU/g DM of various cellulases. ACS (black triangle), cellulase from *T. viride *(open circle), and cellulase from *T. reesei *(black circle). **(C) **Free sugar (sum of glucose, fructose, and sucrose) produced from whole rice plants by the addition of a combination of ACS and glucoamylase. 0.86 FPU/g DM ACS and 3.2 U/g DM glucoamylase (open circle), 0.86 FPU/g DM ACS and 0.32 U/g DM glucoamylase (black square), 0.86 FPU/g DM ACS (black triangle), and without enzyme addition (black circle). Values are expressed as the mean (SD) (n = 3). Values with different lowercase letters are significantly different (*P *< 0.05) as determined by analysis of variance. ACS: cellulase from *Acremonium cellulolyticus*; DM: dry matter; FPU: filter paper unit.

To confirm the feasibility of this method, we analyzed the degradability of biomass and ethanol production in solid-state cellulosic biomass fermentation using a laboratory model system for ensiling. Various whole plants were dried, ground, and sterilized and then used to determine the effects of additives in the process. To investigate the effects of enzymes that degrade structural and nonstructural carbohydrates in solid-state fermentation, cellulase with or without glucoamylase was added to the sterilized whole-plant powder with 60% moisture content. Commercially available ACS was added at a concentration 0.25 to 14.5 times higher than that recommended as a silage additive. The addition of 0.086 FPU/g DM and 0.86 FPU/g DM of ACS to rice whole-plant powder yielded 51 g/kg DM and 131 g/kg DM of free sugar (sum of glucose, fructose, and sucrose), respectively, after 20 days of incubation at 28°C (Figure 2A).

Next we compared the saccharification activity of ACS with commercially available cellulases obtained from *Trichoderma reesei *and *T. viride*. Twenty days after the addition of 0.86 FPU/g DM, higher saccharification activity was observed with ACS and cellulase from *T. viride *compared with cellulase from *T. reesei *(Figure 2B). The mixture of cellulase with other enzymes (pectinase, β-glucosidase, and amylase) induces multiple hydrolyzing activities that function to degrade the fibrous cell wall structure and release starch granules, allowing them to be accessible to amylolytic activity [6,12,13]. ACS and the cellulase from *T. viride *provided relatively higher glucoamylase activity compared with cellulase from *T. reesei *(Table 1). Whole rice plants contain starch not only in the grains, but also in the leaves and stems. We previously reported that incubation with 8.6 FPU/g DM of ACS in 10% (w/v) powdered rice whole crop silage, in 0.1 M sodium acetate buffer (pH 4.5) at 50°C, degraded 6.6% of cellulose, 3.9% of hemicellulose, and 16.6% of starch within three days [6]. These data suggested that the degradation of starch stored in the plant stem and grain would be the major sugar sources for saccharification by ACS and cellulase from *T. viride*. Utilization of starch in the plant body is important to increase ethanol production.

**Table 1:** Comparison of specific activities of the enzymes used in this study.

Specific activity (U/g)	Commercially available enzymes			
	**ACS**	***T. reesei*** **cellulase**	***T. viride*** **cellulase**	***Rhizopus *** **sp. glucoamylase**

Cellulase FPU	261	138	250	NT
Glucoamylase	27	0	12	970
α-glucosidase	36	1	0	7

ACS: cellulase from *Acremonium cellulolyticus*; FPU: filter paper units; NT: not tested.

We then analyzed the effect of glucoamylase in the reaction to observe the further saccharification effect of starch. Adding a combination of 0.32 U/g DM or 3.2 U/g DM of glucoamylase with 0.86 FPU/g DM of ACS to sterilized whole-plant powder with 60% moisture content yielded 182 g/kg DM and 240 g/kg DM of sugar (Figure 2C), respectively. This translates to an increase in the amount of released sugar of about 39% and 83%, respectively, compared with the addition of ACS alone. The maximum ethanol yield, based upon extracted sugar obtained at 20 days after treatment of whole rice plants, with ACS alone (0.86 FPU/g DM) was calculated as 66.8 g/kg DM. In comparison, the yield using a combination of ACS (0.86 FPU/g DM) and glucoamylase (0.32 U/g DM) would be 92.8 g/kg DM.

### Ethanol production from whole plants in a solid-state fermentation model system

**Figure 3:**
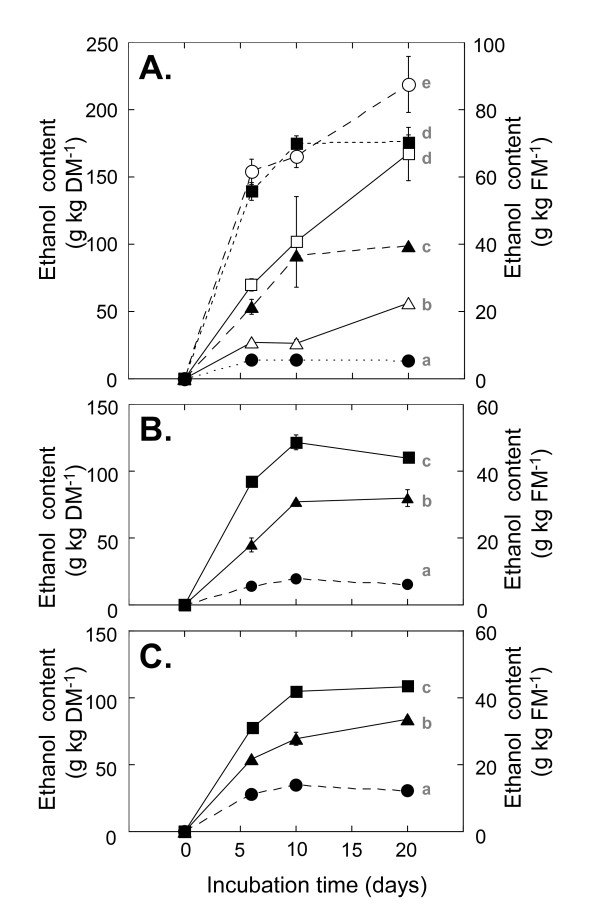
**Ethanol production from various whole plants in solid-state fermentation model system**. Ethanol production from **(A) **whole rice, **(B) **corn, and **(C) **wheat plants with the addition of 0.86 FPU/g DM ACS and 3.2 U/g DM glucoamylase (open circle), 0.86 FPU/g DM ACS and 0.32 U/g DM glucoamylase (black square), 0.086 FPU/g DM ACS and 0.32 U/g DM glucoamylase (open square), 0.86 FPU/g DM ACS (black triangle), 0.086 FPU/g DM ACS (open triangle), and without any enzyme addition (black circle). Values are expressed as the mean (SD) (*n *= 3). Values with different lowercase letters are significantly different (*P *< 0.05) as determined by an analysis of variance. ACS: cellulase from *Acremonium cellulolyticus*; DM: dry matter; FPU: filter paper unit.

To test the accuracy of these theoretical yields, we measured ethanol conversion in materials as described above, but with yeast added at the beginning of the process. After 20 days of incubation at 28°C, the addition of ACS (0.86 FPU/g DM) alone yielded 98.8 g/kg DM of ethanol, whereas a combination of 0.86 FPU/g DM of ACS and 0.32 U/g DM of glucoamylase yielded 176.2 g/kg DM of ethanol (Figure 3A). The actual amounts of ethanol produced were 147% and 187%, respectively, of the theoretical yields, derived from the amount of saccharification by the enzymatic reaction. This discrepancy in the actual and theoretical yields may be caused by the decrement of the final product (sugar) and its inhibitory effect on saccharification. These results confirm the favorable SSF reaction in the solid-state fermentation model system.

Further addition of 11.6 U/g DM of β-amylase, which degrades raw starch, provided a 7% increase in the ethanol yield compared to the addition of 0.86 FPU/g DM of ACS with 0.32 U/g DM of glucoamylase (data not shown). Considering the cost of enzymes, however, in the following experiments with rice we tested only ACS and glucoamylase in combination. To reduce the amount of enzyme required, we tested the effect of one-tenth the amount of ACS. When ACS was decreased to 0.086 FPU/g DM, 56.1 g/kg DM (with ACS alone) and 167.1 g/kg DM (with a combination of ACS and glucoamylase) of ethanol were produced. Thus, by using only 0.086 FPU/g of ACS, ethanol production was reduced to 57% of that yielded by 0.86 FPU/g DM. With the addition of 0.32 U/g DM of glucoamylase and 0.086 FPU/g DM of ACS, ethanol production was 94.8% of that yielded by 0.86 FPU/g DM. These results indicate that in the presence of 0.32 U/g DM of glucoamylase, the addition of 0.086 FPU/g DM of ACS is sufficient for starch saccharification in the solid-state fermentation model system, but the degradation of the plant structure would be increased by the addition of ACS at a higher concentration.

Next, we compared the ethanol production when rice, corn, or wheat plants were used in the model system (Figures 3A-C). The amounts of ethanol produced by the addition of 0.86 FPU/g DM of ACS alone were similar among the three crops. When 0.32 U/g DM of glucoamylase was combined with cellulase, however, ethanol production in the rice treatment was significantly higher than that in the corn or wheat treatments. Further addition of β-amylase with ACS and glucoamylase increased the amount of sugar released from corn plants (data not shown), indicating that the combination of enzymes is important for optimizing the ethanol production from different materials. The average initial pH of the solid-state fermentation model system of rice, corn, and wheat was pH 4.50, pH 4.67, and pH 4.58, respectively, due to the buffering activity of the plant materials. The final pH of all samples was maintained between pH 4.36 and pH 4.57 (data not shown). According to data from the suppliers, the respective optimal pH ranges of ACS and glucoamylase are pH 4.0 to pH 5.0 and pH 4.0 to pH 7.0, meaning that the saccharification process was probably not inhibited. We confirmed that the SSF of the whole rice crop of 10% w/v at 30°C was not inhibited by the presence of up to a 0.7 wt.% of lactic acid between pH 4.23 and pH 4.68, but ethanol production was strongly inhibited at pH 3.96 (data not shown). Similarly, Jing *et al*. reported that the saccharification by cellulase and fermentation by lactic acid bacteria of peashrub woody biomass of 7.3% w/w, at 50°C, was inhibited at pH 3.8 [12]. However, our data indicate that, in the solid-state culture, pH is maintained within an optimum range, and SSF is not inhibited in the presence of 2 wt.% of lactic acid.

### Ethanol production from nonsterilized whole rice plants in laboratory-scale solid-state fermentation

**Figure 4:**
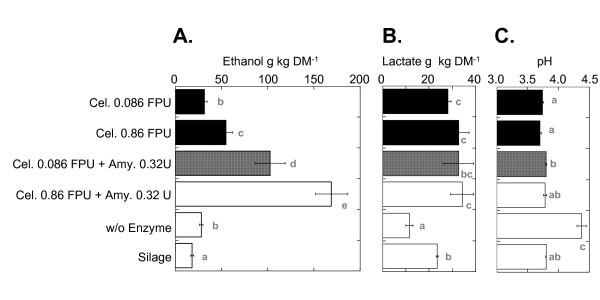
**Evaluation of solid-state fermentation of nonsterilized whole rice plants**. Fermentation was performed for 20 days at 28°C. Ethanol and lactic acid contents and the pH in the fermented products are displayed. Values are expressed as the mean (SD) (*n *= 3). Different lowercase letters following the means in each treatment indicate a significant difference (*P *< 0.05) as determined by an analysis of variance.

To investigate the practicality of the solid-state fermentation process, we performed small-scale solid-state fermentation (250 g wet biomass) using whole nonsterilized forage paddy rice plants containing 62% water (Figure 4). The addition of ACS (0.86 FPU/g DM) and glucoamylase (0.32 U/g DM) yielded 6.4 wt.% fresh matter (FM) of ethanol (equivalent to 169 g/kg DM of ethanol) after 20 days of incubation at 28°C (Figure 4A). When the fermented material was prepared with 0.086 FPU/g DM of ACS and 0.32 U/g DM of amylase, 3.9 wt.% FM (equivalent to 102.9 g/kg DM of ethanol) was produced.

In the solid-state fermentation model system, in the presence of 0.32 U/g DM of glucoamylase, the 0.086 FPU/g DM of ACS treatment produced nearly the same ethanol yield (94.8%) as the 0.86 FPU/g DM treatment. In the laboratory-scale solid-state fermentation system, in the presence of 0.32 U/g DM of glucoamylase, using 0.086 FPU/g DM of ACS produced only 60.9% of the ethanol yield of the 0.86 FPU/g DM treatment. These data suggest the enzymatic degradation of freshly harvested, chopped plant material requires a greater amount of ACS than that for mechanically powdered plant material, which was used in the solid-state fermentation model system. After fermentation, the lactic acid contents of all the laboratory-scale solid-state fermentation products were similar, but they were higher than silage prepared from the same material (Figure 4B). These findings indicate that, in laboratory-scale solid-state fermentation, the lactic acid fermentation also reached its maximum by using the released sugar, and the remaining released sugar may be utilized for ethanol fermentation. The pH values of the solid-state fermentation product and silage were similar (Figure 4C). Based on the ethanol yield in the solid-state fermentation model system, we assumed that the acidic condition did not affect SSF in laboratory-scale solid-state fermentation.

We attempted to recover the ethanol solution from the fermented product containing 6.4 wt.% FM of ethanol using a laboratory-scale rotary evaporator with indirect incubation of the materials in a glass bottle maintained at 58°C by a water bath. The 133-g sample was composed of 8.5 g of ethanol in the 82.46-g liquid fraction (that is, 10.3 wt.% ethanol solution) and 50.5 g of DM. We collected 40 mL of clear solution containing 15.8 wt.% ethanol by chilling the vapor at 4°C. We calculated that 46% of the total moisture, which contained 74% of the total ethanol, was recovered. However, the collection of ethanol solution was inefficient because the thermal conductivity of solid fermented material is much lower than that of liquid. Development of an efficient technique for ethanol recovery is needed.

### Degradation of the carbon component in solid-state fermentation

**Figure 5:**
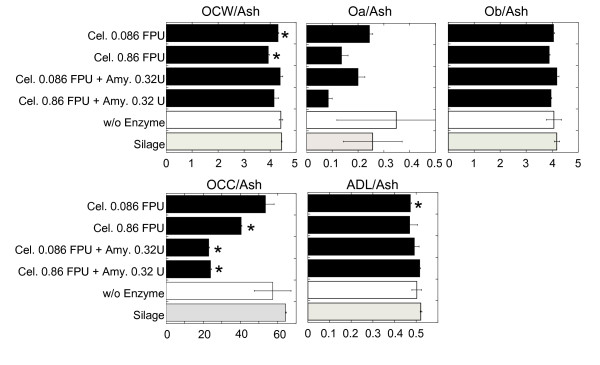
**Effect of various combinations of enzymes on the degradation of fiber and organic cellular content in solid-state fermented whole rice plants**. Forage paddy rice plants were fermented with various enzymes. The relative content of amylase and pronase degradable fraction (Occ) and non degradable fraction (OCW), cellulase degradable fraction of OCW (Oa) and nondegradable fraction (Ob), and ADL compared to the ash content are displayed. Values are expressed as the mean (SD) of two replications. The significance level in the comparison against the silage sample made from the same material was adjusted for the multiplicity effect. *P < 0.05. ADL: acid detergent lignin; Oa: organic a; Ob: organic b; Occ: organic cellular contents; OCW: organic cell wall.

To identify the carbon source for ethanol fermentation, we analyzed the enzymatic degradation abilities of silage and fermented residue made from freshly harvested nonsterilized forage paddy rice plants (Figure 5), following the method for analysis of nutritional value of forage [14,15]. The ratio of lignin (acid detergent lignin, ADL) and ash in each treatment was relatively stable, so we assumed these components remained stable during fermentation. We compared the relative contents of carbon components to the ash content in the solid-state fermented sample treated with cellulase (0.86 FPU/g DM). The relative contents of the amylase and pronase nondegradable fraction, that is, the organic cell wall (OCW) and the amylase and pronase degradable fraction (organic cell contents, Occ) were 12% and 37% less, respectively, than those in the silage. In the sample treated with cellulase (0.86 FPU/g DM) and glucoamylase (0.32 U/g DM), OCW was decreased by 7% and Occ by 63% compared to silage. Glucose derived from saccharification of starch may suppress the simultaneous degradation of fiber. Furthermore, about half the amount of the cellulase degradable fraction (organic a fraction; Oa) was digested during solid-state fermentation, indicating a sufficient effect of cellulase on fiber degradation during fermentation. However, the content of Oa among total fiber in whole rice plants is relatively small, so the decrement of OCW is small compared to that of Occ. Therefore, the Occ, which contains nonstructural carbohydrates, is the important carbon source for ethanol production in solid-state fermentation.

### Analytical nutritional value of solid-fermentation residue

We evaluated the nutritional value of fermented residue as forage (see Table 2 in Additional File 1) compared to silage prepared from the same material and straw of food rice plants (*Koshihikari*). Both silages are commonly used as forage in Japan, so if the chemical composition of fermented residue is comparable to these forages, the material could be used for cattle feed. Generally, fiber is an important component of forage for rumen function and metabolism and is a positive dietary factor. The nutrient value of forage is relatively low compared with other components for cattle feed; greater nutritional value in the form of energy and nitrogen would be favorable. Large decreases in the Occ during solid-state fermentation resulted in a higher lipid content (ether extracts, EE) and greater sum of protein, amino acid, amide, and ammonia (crude protein, Cp) in fermented residue compared with both silages. Amylase treatment during solid-state fermentation induced a strong reduction of the Occ compared with the reduction rate of fiber (acid detergent fiber (ADF) and neutral detergent fiber (NDF)). Therefore, the fiber content and Occ of solid-state fermentation residue is between the values for whole crop silage and rice straw silage. EE, Cp, and fiber are essential diet components for cattle. Thus, the nutritional value of the distilled residue of the solid-state fermentation system should be sufficient for use as feed. Figures 4C and 4B show the pH value and lactic acid contents of the solid-state fermentation products, which have a similar acidic condition and better (higher) lactic acid content than silage prepared from the same material. These findings suggest that the distilled residue and silage would remain free of rot for comparable periods of time.

## Conclusions

SSF was shown to be effective for enzymatic digestibility and fermentability of whole rice plants without any types of sterilized process. It was found that non-structural carbohydrates are the main carbon source for SSF in ethanol and lactic acid fermentation.

The normal bioethanol production system has several problems: bulky materials must be transported to large factories; nutrient loss from biomass and the degradation of materials to sugar occur during preservation; the recovery of ethanol by distillation requires high energy consumption; and the treatment of effluents is necessary. In our system, however, harvested materials are immediately packed into a silo at the field site. Because the fermentation facilities are similar to a conventional silo used for silage fermentation, this system does not require that special facilities be constructed or that bulky materials be transported off site. Immediately after packing harvested materials into the silo, free sugar and enzymatically digestible carbon in the biomass are converted to ethanol and lactic acid during preservation. Although the solid-state fermentation system requires a relatively long time for degradation and fermentation, no energy needs to be supplied to the system; the fermentation period is similar to that of silage fermentation, and farmers are used to preserving silage on their farms. Using a laboratory-scale evaporator, we were able to obtain an approximately 15 wt.% clear ethanol solution by chilling the vapor of the solid fermented biomass. However, the thermal conductivity of the fermented product was very low, which results in wasted energy in evaporating the ethanol solution. After isolation of the ethanol solution, dehydration or distillation of the collected ethanol is easy because it does not contain insoluble particles. Furthermore, the nutritional composition of the fermented residue appears to be suitable for forage, and the residue can be preserved without further treatment. Thus, our findings suggest that the solid-state fermentation of biomass for fuel production can overcome the problems of the normal bioethanol production system. Further studies are needed for a large-scale fermentation system and to evaluate how well the fermented residue can be preserved and digested by cattle. Furthermore, it is necessary to develop a suitable method for isolating ethanol from fermented products near the silo, without consuming a lot of fossil fuel or producing liquid waste.

## Methods

### Solid-state fermentation model system

To prepare the solid-state fermentation model system, whole plants (rice, 'Akihikari'; corn, 'Wasehomare'; wheat, 'Nourin No. 61'; cultivated in Okinawa, Tochigi, and Ibaraki, respectively, in Japan) were dried in an oven (O-190FDS, Sunaka Rika Kogyo, Tokyo, Japan) at 70°C for three days, powdered using a laboratory-scale mill (A11BS1, IKA, Staufen, Germany), passed through 2-mm mesh, and sterilized by electron beam (25.0 kGy) using a 5-MeV dynamitron accelerator (Radiation Dynamics Inc., Edgewood, NY, USA). All chemicals used were analytical grade and obtained from Sigma-Aldrich (St. Louis, MO, USA) or Wako Chemicals (Osaka, Japan). Filter paper degradation activity of saccharifying cellulase (FPU assay) and the activities of glucoamylase and α-glucosidase were measured as described previously [6]. Appropriate amounts of enzymes (ACS, Meiji Seika Pharma, Co., Tokyo, Japan; cellulase from *T. reesei*, GC220, Genencor International Inc., New York, USA; cellulase from *T. viride*, C0615-16, Sigma-Aldrich; glucoamylase from *Rhizopus *sp., Oriental Yeast Co., Tokyo, Japan) were added to 0.5 g of plant powder containing a final concentration of 2% lactic acid and 60% moisture in a 15-mL screw-capped glass tube. Yeast inoculum of *Saccharomyces cerevisiae *IFO0304 was cultivated in 30 mL of 1% yeast extract (Becton Dickinson Co., Sparks, NV, USA), 2% peptone (Becton Dickinson), and 2% glucose in a 300-mL volume Erlenmeyer flask, which was shaken at 200 rpm on a rotary shaker (Thermostatic Shaking Incubator, IALRS310, Thomas Co., Tokyo, Japan) at 30°C for 17 hours. After cultivation, cells were collected by centrifugation and washed with 30 mL of sterilized water. To analyze the ethanol fermentation ability, a freshly cultivated cell suspension of *S. cerevisiae *IFO0304 was inoculated (3 × 10^6^/g FM) in the same system at the beginning of fermentation.

### Laboratory-scale solid-state fermentation system

Forage paddy rice material ('Ushimoe', cultivated at Saitama, Japan) was prepared by mixing chopped straw (3 cm to 5 cm length) from the whole plant with hulls and brown rice separated previously. Appropriate amounts of enzymes (cellulase and glucoamylase), *S. cerevisiae *(3 × 10^6^/g FM), and freeze-dried lactic acid bacteria (Chikuso No. 1 for silage additive, 5 μg/g FM, as recommended by the manufacturer Snowseed Co., Sapporo, Japan) were added. Materials (250 g FM) were mixed with enzymes, *S. cerevisiae*, and lactic acid bacteria and packed with CO_2_-absorbent material (10 portions of C-1001P, Mitsubishi Gas Co., Tokyo, Japan) in a plastic bag lined with aluminum film, and sealed by packing machine that contained a suction pump (V-301, Fuji Impulse, Osaks, Japan). For comparison, silage was prepared from the same material or straw of food rice plant (*Koshihikari*) as above, but with only the addition of lactic acid bacteria.

### Analysis of fermentation products

We prepared three samples for each analytical point, and incubated at 28°C in a low-temperature incubator (IL600, Yamato, Tokyo, Japan). After incubation of the prepared materials of the silage fermentation model system, four times the initial weight of sterilized water was added to each bottle. Laboratory-scaled fermentation were incubated for 20 days and, immediately after opening the pouch, 20 g portions of each of the fermented products were collected in a K-nylon-layered polyethylene bag (Hiryu, Asahi Kasei, Tokyo, Japan), soaked in four times their weight of sterilized water, and sealed by polysealer (P-200, Fuji Impulse). Samples were shaken for 30 minutes at 100 rpm at 20°C, and the extracted solution was used to analyze ethanol, sugar (glucose, fructose, sucrose), and DL- lactic acid contents using Boehringer Mannheim UV-test assay kits (ethanol: code number 10176290036; D-glucose/D-fructose: 10139106035; sucrose: 10139041035; and D-lactic/L-lactic acid: 11112821035; R-Biopharm AG, Darmstadt, Germany). In every assay, we also analyzed chemical grade materials as the standard. The nutrient values of silage and fermented products were analyzed following the protocol for forage analysis [10,14,15]. Chemical analysis for the content of ash, ADL, EE, Cp, ADF, and NDF were performed as described previously [10]. Enzymatically digestible components were analyzed as described previously [14,15], as follows: organic matter was divided into amylase and pronase degradable fractions (Occ) and nondegradable fraction (OCW), and the cellulase-degradable fraction of OCW (Oa) and nondegradable fraction (organic b) were analyzed.

### Ethanol collection from solid-state fermented whole rice plants

An ethanol recovery test was performed using a laboratory-scale rotary evaporator (RE121, Shibata, Tokyo, Japan), with incubation in a water bath (461, Shibata) at 58°C, and a hand-held aspirator (WP-11, Yamato). Vapor was collected at 4°C, maintained using a coolant bath (EL15-F, Taitec, Tokyo, Japan). The 133 g of fermented material was divided into three 30-g samples and a 43-g sample, and each sample was evaporated for 30 minutes. The total ethanol solution collected from all samples was analyzed.

## Competing interests

The authors declare that they have no competing interests.

## Authors' contributions

HKK designed the research and wrote the manuscript; MH collected data; YS assisted in the analysis of fermented products; YC analyzed the nutritional contents; and KS collected the ethanol. All authors read and approved the final manuscript.

## Supplementary Material

Additional file 1 **Table 2**. Nutritional contents in fermentation residue of solid-state whole rice plants. Click here for file
