# Supplementary material for: Silage produces biofuel for local consumption
Source: Biotechnol Biofuels. 2011 Nov 1;4:46. doi: 10.1186/1754-6834-4-46 (PMC3224754; doi:10.1186/1754-6834-4-46)
Supplement: Additional file 1 — Table 2. Nutritional contents in fermentation residue of solid-state whole rice plants. [file 1754-6834-4-46-S1.DOC]

Table 2. Nutritional contents in fermentation residue of solid-state whole rice plants

|  | ADL | | | | EE | | | | Cp | | | | ADF | | | | NDF | | | | Occ | | | |
| --- | --- | --- | --- | --- | --- | --- | --- | --- | --- | --- | --- | --- | --- | --- | --- | --- | --- | --- | --- | --- | --- | --- | --- | --- |
|  | Av. | SD | a | b | Av. | SD | a | b | Av. | SD | a | b | Av. | SD | a | b | Av. | SD | a | b | Av. | SD | a | b |
| Cel.0.086 FPU | 4.46 | 0.10 |  |  | 1.96 | 0.01 |  | ** | 5.90 | 0.05 |  | ** | 23.42 | 1.30 |  | ** | 37.11 | 9.05 |  | ** | 50.09 | 10.87 |  | ** |
| Cel.0.86 FPU | 5.23 | 0.09 |  |  | 2.16 | 0.00 | * | ** | 6.14 | 0.02 |  | ** | 25.48 | 0.68 | * | ** | 41.08 | 2.94 |  | ** | 45.07 | 2.02 | * | ** |
| Cel.0.086 FPU + Amy. 0.32U | 6.40 | 0.04 | * | ** | 2.28 | 0.00 | * | ** | 6.26 | 0.02 | * | ** | 33.21 | 0.71 | * | | 53.76 | 2.72 | * |  | 29.83 | 6.09 | * | ** |
| Cel.0.86 FPU + Amy. 0.32U | 6.86 | 0.01 | * | ** | 2.96 | 0.00 | * | ** | 8.20 | 0.02 | * | ** | 30.69 | 0.01 | * | ** | 51.54 | 0.00 | * | ** | 31.71 | 9.40 | * | ** |
|  |  |  |  |  |  |  |  |  |  |  |  |  |  |  |  |  |  |  |  |  |  |  |  |  |
| w/o Enzyme | 4.53 | 0.34 |  |  | 1.97 | 0.01 |  | ** | 5.92 | 0.10 |  | ** | 23.62 | 5.57 |  | ** | 37.75 | 20.13 |  | ** | 51.22 | 21.70 |  | ** |
| Silage | 4.40 | 0.01 | - | ** | 1.77 | 0.00 | - |  | 5.77 | 0.00 | - | ** | 22.17 | 0.10 | - | ** | 37.33 | 0.52 | - | ** | 54.18 | 0.53 | - | ** |
|  |  |  |  |  |  |  |  |  |  |  |  |  |  |  |  |  |  |  |  |  |  |  |  |  |
| Rice straw silage | 5.34 | 0.07 | * | - | 1.62 | 0.00 |  | - | 2.43 | 0.00 | * | - | 34.06 | 0.04 | * | - | 58.08 | 1.09 | * | - | 19.82 | 4.60 | * | - |

Forage paddy rice plants were fermented with various enzymes. Contents of acid detergent lignin (ADL), ether extracts (EE), and crude protein (Cp), acid detergent fibre (ADF), neutral detergent fibre (NDF), and organic cellular contents (Occ), and were analyzed. Values are expressed as the mean (SD) of two replicates. The significance levels in the comparisons against silage samples prepared from the same forage paddy rice plants (column a; *) and straw of food rice plants (column b; **) were adjusted for the multiplicity effect. *P*<0.05, -: not tested
